# Arbuscular mycorrhizal fungi in a wetland constructed for benzene-, methyl *tert*-butyl ether- and ammonia-contaminated groundwater bioremediation

**DOI:** 10.1111/j.1751-7915.2012.00357.x

**Published:** 2012-07-31

**Authors:** Thomas Fester

**Affiliations:** Helmholtz-Centre for Environmental Research – UFZPermoserstraße 15, D-04318, Leipzig, Germany

## Abstract

Arbuscular mycorrhizal fungi (AMF), which are present in most natural environments, have demonstrated capacity to promote biodegradation of organic pollutants in the greenhouse. However, it is not certain whether AMF can spontaneously establish in phytoremediation systems constructed to decontaminate groundwater, because of the unusual conditions during the construction and operation of such systems. To assess this possibility, root samples from a wetland constructed for the phytoremediation of groundwater contaminated with benzene, methyl *tert*-butyl ether and ammonia were analysed. Substantial AMF colonization was observed in plant roots sampled close to the inlet of a basin filled with fine gravel and planted with *Phragmites australis*. In addition, analysis of a fragment of the nuclear large ribosomal subunit, amplified by nested PCR, revealed the presence of AMF molecular operational taxonomic units closely related to *Funneliformis mosseae* and *Rhizophagus irregularis* in the samples. These findings demonstrate the capacity of generalist AMF strains to establish spontaneously, rapidly and extensively in groundwater bioremediation technical installations.

## Introduction

An arbuscular mycorrhiza is a type of close, mutualistic association that forms in root systems between diverse plant species and members of a small group of soil fungi (arbuscular mycorrhizal fungi, AMF; Smith and Read, 2008). The association allows the exchange of nutrients (carbohydrates provided by the plant, mineral nutrients provided by the fungi), and markedly increases the host plant's tolerance of various biotic and abiotic stress factors. Arbuscular mycorrhizal fungi also influence the transport and distribution of organic pollutants in plants (Debiane *et al*., 2009; Langer *et al*., 2010), reportedly reducing their concentrations in shoots of colonized plants, while increasing their concentrations in roots, particularly in the rhizodermis (Huang *et al*., 2007; Wu *et al*., 2009). These effects may help to protect plants from damage by organic pollutants. Beneficial effects of the presence of AMF on soil bacteria (Toljander *et al*., 2007), notably bacteria capable of degrading organic compounds (Corgié *et al*., 2006; Alarcon *et al*., 2008), have also been reported. By both protecting plants from adverse effects of organic pollutants and promoting associated bacteria, AMF can accelerate the biodegradation of organic pollutants. Several studies have recently demonstrated beneficial effects of AMF on the biodegradation of organic pollutants, including: the dissipation of polycyclic aromatic hydrocarbons (PAHs) by *Lolium multiflorum* (Yu *et al*., 2011), dissipation of PAHs by *Medicago sativa* under low water and phosphate availability (Zhou *et al*., 2009), and phytoremediation of aged petroleum contamination by *Triticum aestivum* (Malachowska-Jutsz and Kalka, 2010). Arbuscular mycorrhizal fungi can therefore be considered ideal inhabitants of technical installations for the plant-based bioremediation of groundwater contaminated by organic pollutants. However, such installations are often constructed without including a significant source of AMF propagules. Furthermore, the stressful conditions in such installations – such as poor substrates, and potentially toxic concentrations of organic pollutants for the fungi (Verdin *et al*., 2006; Debiane *et al*., 2011) – may hinder the successful establishment of AMF.

To investigate the ability of AMF to establish under such conditions, we analysed AMF colonization levels in plant roots sampled from a wetland constructed to decontaminate groundwater polluted with benzene, methyl *tert*-butyl ether (MTBE) and ammonia. The wetland was continuously streamed (inflow rate 6 l h^−1^) by water containing 20, 3.7 and 45 mg l^−1^ of these compounds respectively. Arbuscular mycorrhizal fungi present in roots from *Phragmites australis* growing in this wetland were phylogenetically analysed by cloning and sequencing a 400 bp fragment of the nuclear large ribosomal subunit, amplified by nested PCR.

## Results and discussion

### Spontaneous colonization of constructed wetlands

**Fig. 1:**
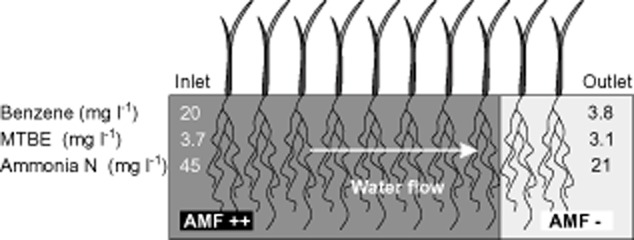
In March 2011 five samples of roots (each about 10 g) were taken from the ‘front’ (near the inlet) and five from the ‘rear’ (near the outlet; 10 samples in total) of the illustrated constructed wetland (5 m long, 1.15 m wide, 1.25 m deep; inflow rate 6 l h^−1^) planted with *P. australis*, which is being used in a compartment transfer experiment close to Leuna, Germany (Seeger *et al*., 2011). Parts of the sampled roots were stained with ink (Sheaffer, Middlesex, UK) and vinegar according to Vierheilig and colleagues (1998) to highlight AMF structures, and the degree of colonization by AMF was roughly estimated by inspecting the stained roots under a stereomicroscope and estimating approximate ratios of mycorrhizally colonized to non-colonized root lengths. Substantial degrees of AMF colonization were observed in all five root samples from the ‘front’ part of the wetland (40%, 25%, 25%, 60% and 80%). In contrast, no colonization of *P. australis* roots was observed in samples from the rear part, where there was no gravel substrate and the roots formed a dense root mat. These microscopic observations are consistent with results of nested PCR analysis of a 400 bp fragment of the nuclear large ribosomal subunit using the primer pairs LR1/FLR2 and FLR3/FLR4 (Gollotte *et al*., 2004) and Taq PCR Mastermix (Qiagen, Hilden, Germany). DNA extracted (using a DNeasy Plant Mini-Kit, Qiagen) from all samples from the front part of the wetland yielded fragments of expected size (for AMF), while DNA extracted from samples from the rear part yielded no PCR products. The concentrations of pollutants (benzene, methyl *tert*-butyl ether/MTBE and ammonia N) shown in the figure have been taken from Seeger and colleagues (2011).

The constructed wetland investigated in this study was established in March 2007. It consists of a basin that receives a stream of contaminated groundwater. *Phragmites australis* plantlets were planted at the inlet end, which is filled with light gravel. Close to its outlet area there is a compartment lacking the gravel substrate where *P. australis* is growing in water, forming a dense root mat (Fig. 1). Root samples taken from the part of the constructed wetland with the gravel substrate in 2011 were substantially associated with AMF (colonized proportions by length, 40%, 25%, 25%, 60% and 80%; see Fig. 1 legend for details), clearly showing that these fungi successfully colonized this unusual environment within 4 years. Thus, establishment of AMF does not appear to have been profoundly hindered in the inlet part of the wetland, although it was exposed to the highest concentrations of organic pollutants. In contrast, no colonization of roots by AMF was observed in the part of the basin where the plants were growing in free water with no gravel substrate, suggesting that a solid substrate was required for AMF colonization. The likeliest sources of the colonizing fungi were airborne propagules or mycelia already present in the *P. australis* plantlets when they were transferred to the constructed wetland.

### Generalist AMF strains as early and rapid colonizers of the constructed wetland

**Fig. 2:**
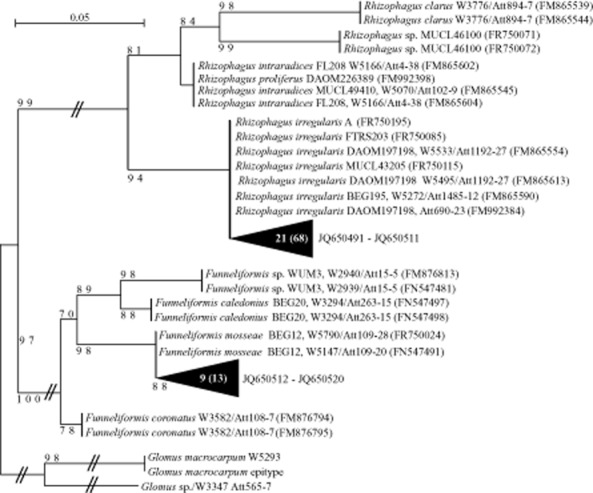
PCR amplification products of a fragment of the nuclear large ribosomal subunit obtained from DNA in root samples collected from the front part of the constructed wetland were purified (using a peqGold Cycle-Pure Kit; Peqlab, Erlangen, Germany), pooled and ligated into pCR 2.1 (using a TA cloning kit; Life Technologies, Darmstadt, Germany). After transformation into *Escherichia coli* DH5α, 85 positive clones were identified using classical blue–white screening, and amplified by colony PCR (using M13 primers). The resulting products were screened by Taq I restriction digestion at 37°C overnight, and analysis of electrophoretic patterns using GelCompar II (Applied Maths NV, Sint-Marten-Latern, Belgium). Forty-eight clones with identical restriction patterns were identified and eliminated from further analysis. PCR products from the remaining 34 clones were purified using a SureClean kit from Bioline (Luckenwalde, Germany) and sequenced using a BigDye® Terminator v3.1 Cycle Sequencing Kit (Applied Biosystems, Foster City, USA) and a 3130xl Genetic Analyzer (Applied Biosystems). Sequences were edited (by removing primer and vector sequences, and controlling sequence quality) using Sequencher 4.8 (Gene Codes Corporation, USA). Database searches for similar sequences were performed using the blast program (Altschul *et al*., 1990). In a few cases, non-glomeromycotan sequences (similar to sequences from the basidiomycotan genus *Cryptococcus*) were found. The ClustalW2 algorithm implemented in Seaview (Gouy *et al*., 2010) was used to align sequences with corresponding sequences from AMF strains defined in Krüger and colleagues (2011). Most of these sequences refer to individual GenBank accessions, although the sequences for *Glomus* sp. W3347/Att565-7, *Glomus macrocarpum* W5293 and *G. macrocarpum* epitype refer to consensus sequences defined in Krüger and colleagues (2011). Seaview was also used to construct neighbour joining trees (using BioNJ and Kimura 2-parameter models, with 1000 bootstrap permutations) and the maximum likelihood tree shown here (model: general time reversible, starting from a neighbour joining/BioNJ tree, with branch support estimated using the approximate likelihood ratio test approach). The genus *Glomus* was used as an out-group in this tree. Branches were collapsed to those branches showing unique Taq I restriction patterns in a virtual digest. GenBank accession numbers for the sequences obtained in this study (black triangles) are shown. Numbers within the triangles refer to the numbers of respective sequences analysed and to numbers of clones with concordant Taq I digestion patterns (in brackets).

Considerable frequencies of very similar patterns were detected in restriction fingerprinting of PCR products cloned from a fragment of the large ribosomal subunit, indicating that the AMF community within the constructed wetland had low diversity at the sampling time. Fifty-one clones with identical patterns were removed from the analysis, leaving 34 unique clones for sequence analysis, and only two AMF taxa were detected: *Rhizophagus irregularis* and *Funneliformis mosseae*. The restriction endonuclease Taq I was used for restriction fingerprinting, partly because it has been recommended for T-RFLP analysis of the PCR fragment analysed in this study (Mummey and Rillig, 2007), and partly because almost all AMF species in the phylogenetic tree shown in Fig. 2 could be differentiated using this enzyme in a virtual digest. In particular, it was possible to differentiate all other species from *R. irregularis* and *F. mosseae*, the two AMF found in the wetland samples, excluding the possibility that AMF species were missed because of the use of Taq I for restriction fingerprinting prior to sequence analysis. As the primer pairs used in our analysis are not capable of amplifying sequences of members from the genus *Diversispora* or the families *Archaeosporaceae* and *Paraglomaceae* (Gamper *et al*., 2009), however, the possible presence of additional AMF from these groups cannot be excluded. Phylogenetic analysis using the set of consensus sequences for AMF (see fig. 1 in Krüger *et al*., 2011) clearly showed that all sequences analysed in our study clustered with the AMF genera *Rhizophagus* or *Funneliformis* (data not shown). Only four sequences clustered with different fungal groups, one of which proved to be a chimeric sequence in later analysis, while the other three were very similar to sequences of the basidiomycotan genus *Cryptococcus*. We have previously observed unspecific amplification of nuclear rRNA from this genus using the primers FLR3 and FLR4 on a number of occasions. *Cryptococcus* is a large fungal genus with some species that are pathogenic for humans. Although substrate (light gravel) and inflowing water (contaminated groundwater) can be expected to be relatively poor inocula in general, introduction of members from *Cryptococcus* by these sources cannot be excluded. Alternatively, airborne spores have been described for the pathogenic species (Hajjeh *et al*., 1995; Kidd *et al*., 2007) and appear also possible as sources of inoculation in the case presented here.

To examine the sequences clustering with *Rhizophagus* or *Funneliformis* in more detail, all reference sequences not belonging to either of these genera or the out-group genus *Glomus* were removed, while more sequences from *Rhizophagus* and *Funneliformis* – summarized by Krüger and colleagues (2011) – were included in the analysis. After sequence alignment and construction of a maximum likelihood tree (using the general time reversible evolutionary model), the sequence groups clustering with *Funneliformis* showed a close relationship with *F. mosseae*, while those clustering with *Rhizophagus* clustered exclusively with sequences from *R. irregularis* (Fig. 2). The phylogenetic tree produced from the applied sequences (Fig. 2) was therefore restricted to those branches in *R. irregularis* and *F. mosseae* that can be differentiated by virtual digestion using Taq I. As already mentioned, the possible presence of additional AMF from the *Diversispora*, *Archaeosporaceae* or *Paraglomaceae* cannot be excluded, because of limitations of the primers used in our analysis. Nevertheless, the observation only of sequences connected to *R. irregularis* and *F. mosseae*, after screening 85 and sequencing 34 sequences, corroborates the preliminary indications that the constructed wetland contained an AMF community with very low diversity.

*Rhizophagus irregularis* refers to a large part of the taxonomic group that was previously known as *Glomus intraradices*, while *F. mosseae* was previously named *Glomus mosseae* (Schüßler and Walker, 2010). Both of these species are known to be typical generalist AMF (Öpik *et al*., 2006; Rosendahl *et al*., 2009; Oehl *et al*., 2010) that have been found in diverse habitats around the world. Although they have not been mentioned specifically in analyses of AMF succession (Piotrowski and Rillig, 2008), they seem to be pioneer AMF strains in the constructed wetland we studied.
